# Synthesis of Novel Temperature- and pH-Sensitive ABA Triblock Copolymers P(DEAEMA-*co*-MEO_2_MA-*co*-OEGMA)-*b*-PEG-*b*-P(DEAEMA-*co*-MEO_2_MA-*co*-OEGMA): Micellization, Sol–Gel Transitions, and Sustained BSA Release

**DOI:** 10.3390/polym8110367

**Published:** 2016-11-11

**Authors:** Yanan Han, Shouxin Liu, Hongguang Mao, Lei Tian, Wenyan Ning

**Affiliations:** Key Laboratory of Applied Surface and Colloid Chemistry, Ministry of Education, School of Chemistry and Chemical Engineering, Shaanxi Normal University, Xi’an 710062, China; Hanyanan0603@163.com (Y.H.); mhg1130@163.com (H.M.); 18729349848@163.com (L.T.); 18792866293@163.com (W.N.)

**Keywords:** temperature- and pH-sensibility, micellization, sol–gel transitions

## Abstract

Novel temperature- and pH-responsive ABA-type triblock copolymers, P(DEAEMA-*co*-MEO_2_MA-*co*-OEGMA)-*b*-PEG-*b*-P(DEAEMA-*co*-MEO_2_MA-*co*-OEGMA), composed of a poly(ethylene glycol) (PEG) middle block and temperature- and pH-sensitive outer blocks, were synthesized by atom transfer radical polymerization (ATRP). The composition and structure of the copolymer were characterized by ^1^H NMR and gel permeation chromatography (GPC). The temperature- and pH-sensitivity, micellization, and the sol–gel transitions of the triblock copolymers in aqueous solutions were studied using transmittance measurements, surface tension, viscosity, fluorescence probe technique, dynamic light scattering (DLS), zeta-potential measurements, and transmission electron microscopy (TEM). The lower critical solution temperature (LCST) of the triblock copolymer, which contains a small amount of a weak base group, (*N*,*N*-diethylamino) ethyl methacrylate (DEAEMA), can be tuned precisely and reversibly by changing the solution pH. When the copolymer concentration was sufficiently high, increasing temperature resulted in the free-flowing solution transformation into a micellar gel. The sol-to-gel transition temperature (*T*_sol–gel_) in aqueous solution will continue to decrease as solution concentration increases.

## 1. Introduction

While chemically cross-linked polymer gels are being widely used and continuously evaluated, there is a growing interest in stimuli-responsive block copolymer aqueous micellar gels (namely, physically cross-linked gels) [1,2,3,4,5,6,7,8]. These physically cross-linked gels, responsive to temperature, pH, light, salt, and magnetic or electric fields have received considerable attention [8,9,10,11,12,13,14,15]. Compared with chemically cross-linked hydrogels, these responsive micellar gels, especially those induced by temperature or pH, can be more advantageous for extensive applications because of their biocompatibility and pharmaceutical applications. The lower critical solution temperature (LCST) of the thermoresponsive polymer, poly(*N*-isopropylacrylamide) (PNIPAM), was about 32 °C in aqueous solution. Therefore, PNIPAM has been used frequently as a thermoresponsive polymer in recent years [15,16,17,18].

However, PNIPAM has many drawbacks. For instance, PNIPAM has a single LCST at 32–33 °C and does not have biocompatibility. Therefore, researchers are exploring some new thermoresponsive polymers. The copolymer (P(MEO_2_MA-*co*-OEGMA)—composed of 2-(2-methoxyethoxy) ethyl methacrylate (MEO_2_MA) and oligo (ethylene glycol) methacrylate (OEGMA)—has been proposed as an interesting alternative to PNIPAM. When Lutz and coworkers changed the ratio of OEGMA and MEO_2_MA, a sharp and tunable LCST in water (between 26 and 90 °C) was attainable [19,20,21]. Compared with PNIPAM, a great advantage of this thermoresponsive copolymer lies in the fact that it is mainly composed of biocompatible oligo (ethylene glycol) segments which can be widely used in biomedical and biotechnology applications, and its tunable LCST compared to the single LCST of PNIPAM at 32–33 °C. In addition to PNIPAM and P(MEO_2_MA-*co*-OEGMA), polypropylene glycol (PPG) is another type of thermoresponsive material that has been fabricated into thermogels, micelles, and so forth. This sophisticated polymer has been widely explored in many applications including drug delivery, protein protection, and rheology modifier [22,23,24,25,26].

Poly(ethylene glycol) (PEG) is a cheap, neutral, hydrophilic, biocompatible, FDA-approved synthetic polymer that has a wide range of applications in biotechnology and medicine [27]. PEG in hydrogels can enhance water-swelling ability, hydrogel permeability, and solute release [28,29,30,31,32]. PEG as a hydrophilic building block has been extensively used as a constituent in a variety of functional polymers. For instance, PEG is an excellent shielding agent for in vivo delivery of various bioactive compounds. Thus, PEG is extensively used in delivery of either low molecular weight drugs, active peptides, proteins, or even genetic material [33,34,35].

ABA triblock copolymers, composed of a permanently water-soluble B block and stimuli-responsive hydrophilic A blocks, can self-assemble in aqueous solution. When the concentration of the copolymer in aqueous solution is lower, it forms flowerlike micelles, with the outer blocks associating into the core and the central blocks forming loops in the corona layer upon application of an external stimulus. However, when the copolymer concentration is sufficiently high (above the critical gelation concentration (CGC)), a 3-dimensional micellar network is formed, in which the central block forms bridges among neighboring micelles [8]. Lutz synthesized various linear ABA triblock copolymers (P(MEO_2_MA-*co*-OEGMA)-*b*-PEG-*b*-P(MEO_2_MA-*co*-OEGMA)) and four-arm star-block copolymers (sPEG-*b*-P(MEO_2_MA-*co*-OEGMA)) via atom transfer radical polymerization (ATRP), and studied the reversible thermoresponsive properties in aqueous medium and sol–gel transitions [36].

Nevertheless, thermoresponsive polymers alone cannot satisfy the needs of bioscience. Researchers are interested in copolymers responsive to two or more stimuli [4,7,37,38,39,40,41,42,43]. Among responsive copolymers, temperature- and pH-sensitive copolymer is one of the most popular multi-responsive copolymer. Zhao systematically studied the properties of aqueous micellar gels formed from aqueous solutions of a thermo- and pH-responsive ABA triblock copolymer [8,9,10,11,12]. Moreover, it has been reported that the LCST of a thermo- and pH-responsive copolymer can be reversibly tuned by adjusting the solution pH [44,45,46,47,48,49,50].

Poly (*N*,*N*-diethylamino) ethyl methacrylate (PDEAEMA) is one of the pH-responsive polymers, and is a weak basic polyelectrolyte as well. In aqueous solution, the tertiary amine groups of PDEAEMA are protonated under acidic conditions, which causes the polymer to be typically hydrated, swollen, and hydrophilic in its ionic state. However, the pH-responsive segments are deprotonated under basic condition, which causes PDEAEMA to be dehydrated, compact, and more hydrophobic [51]. According to what we have discussed above, novel temperature- and pH-responsive ABA-type triblock copolymer, P(DEAEMA-*co*-MEO_2_MA-*co*-OEGMA)-*b*-PEG-*b*-P(DEAEMA-*co*-MEO_2_MA-*co*-OEGMA), has been synthesized and is composed of two parts: the hydrophilic PEG as part B, and the thermo- and pH-responsive blocks P(DEAEMA-*co*-MEO_2_MA-*co*-OEGMA) as part A. The key feature of this dual stimuli-responsive hydrophilic ABA triblock copolymer is that its LCST can be modulated by introduction of DEAEMA and OEGMA.

In this work, thermo- and pH-induced sol–gel transitions of the ABA triblock copolymer in aqueous solutions, under moderate concentration, and at various temperatures and pH values were studied. Bovine serum albumin (BSA) is the most abundant globular protein in the plasma. This protein is commonly used as a model protein due to its medicinal importance, low cost, ready availability, stability, water solubility, and structural similarity to human serum albumin (HSA). In this work, sustained BSA release in the ABA triblock copolymer gel was also studied.

## 2. Materials and Methods

### 2.1. Materials

Poly(ethylene glycol) (95%, PEG, *M*_W_ = 2000, 4000, 8000, and 10,000 g·mol^−1^) was dried under high vacuum. Before use in experiments, 2-(2-methoxyethoxy) ethyl methacrylate (MEO_2_MA, 95%, *M*_n_ = 188 g·mol^−1^), oligo(ethylene glycol) methacrylate (OEGMA, 95%, *M*_n_ = 475 g·mol^−1^), and (*N*,*N*-diethylamino) ethyl methacrylate (DEAEMA, 98%, *M*_n_ = 185 g·mol^−1^) (J&K Chemical Technology Co., Ltd., Beijing, China) were purified by distillation. Copper (I) chloride (Cu(I)Cl, 99.999%) (Alfar, Shanghai, China), 2,2-bipyridine (bpy, 99%) and the ATRP initiator 2-bromoisobutyryl bromide (98%, Acros, Beijing, China) were used as received. BSA was obtained from Sigma-Aldrich (Shanghai, China). Toluene was dried with metallic sodium, distilled, and stored in a solvent storage flask. Anhydrous ether, methanol (CH_3_OH) purified by distillation, tetrahydrofuran (THF), and dichloromethane (CH_2_Cl_2_) were purchased from Shanghai Chemical Reagent (Shanghai, China). All other chemicals were purchased from either Shanghai Chemical Reagent or J&K and used without further purification.

### 2.2. Characterization

Gel permeation chromatography (GPC) traces were carried out at ambient temperature using GPC (Waters-Breeze, Manchester, UK). The data were processed using Cirrus GPC software (Malvin, UK). THF was used as the carrier solvent at a flow rate of 1.0 mL·min^−1^. Polystyrene standards were used for calibration. The ^1^H NMR spectra were recorded on a Bruker AVANCE 300 MHz spectrometer (Bruker Corporation, Faellanden, Switzerland). Deuteration chloroform was used as the carrier solvent, and the residual solvent proton signal was used as the internal standard.

### 2.3. Synthesis of Difunctional Macroinitiator (Br–PEG–Br)

Poly (ethylene glycol) (PEG) dried under a high vacuum with a molecular weight of 4000 g·mol^−1^ (1.595 mmol, 6.378 g) was added into a three-necked flask and dissolved in toluene (300 mL). Triethylamine (13.8 mmol, 1.95 mL) was added into the flask under the protection of N_2_, the mixture was stirred for 30 min, then 2-bromoisobutyryl bromide (27.45 mmol, 3.45 mL) was added dropwise within 40 min. After the mixture was stirred overnight at ambient temperature, the precipitate was removed by vacuum filtration. The filtrate was concentrated through rotary evaporation. Then, the viscous solution obtained from the previous step was purified by precipitation in diethyl ether (three times) from its dichloromethane solution. The macroinitiator was then dissolved in distilled water and the dichloromethane was used to extract the Br–PEG–Br. Then, the dichloromethane solution of macroinitiator was purified over anhydrous magnesium sulfate overnight. After the removal of magnesium sulfate by filtration, and the concentration of filtrate through rotary evaporation, the viscous solution was precipitated in diethyl ether. Lastly, the precipitation was dried under high vacuum at ambient temperature. Then, the macroinitiator was obtained as a white solid, and it was named Br–PEG–Br2. Furthermore, Br–PEG–Br1, Br–PEG–Br3, and Br–PEG–Br4 were synthesized with PEG of molecular weights of 2000, 8000, and 10,000 g·mol^−1^ respectively according to the above method [8].

### 2.4. Synthesis of ABA Triblock Copolymer P(DEAEMA-co-MEO_2_MA-co-OEGMA)-b-PEG-b-P(DEAEMA-co-MEO_2_MA-co-OEGMA)

**Scheme 1:**
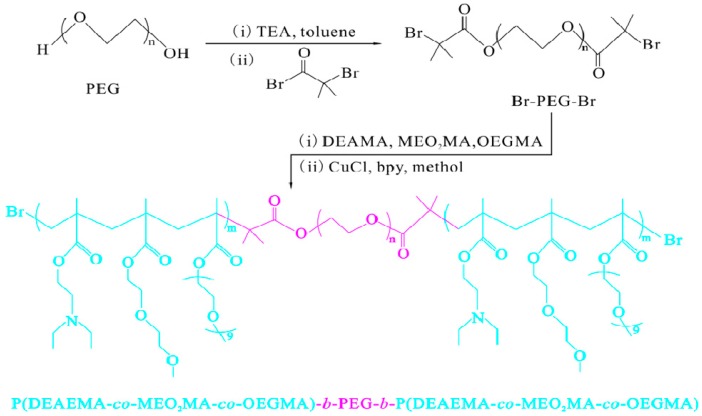
Schematic illustration of the synthesis of ABA triblock copolymer P(DEAEMA-*co*-MEO_2_MA-*co*-OEGMA)-*b*-PEG-*b*-P(DEAEMA-*co*-MEO_2_MA-*co*-OEGMA).

The macroinitiator Br–PEG–Br2 (0.0538 mmol, 0.125 g) along with methanol (5 mL) were added to a Schlenk tube (reaction tube), and when Br–PEG–Br2 was sufficiently dissolved in methanol, MEO_2_MA (9.5 mmol, 1.8 mL), DEAEMA ddeded DEAEMA (0.7527 mmol, 149 µL), and OEGMA (0.5 mmol, 222 µL) (*n*_(MEO2MA__)_:*n*_(DEAEMA__)_:*n*_(OEGMA__)_ = 38:3:2) were added. Then, the mixture was stirred for 30 min under a protection of N_2_ atmosphere. Three freeze-pump-thaw cycles were employed to degas the reaction mixture. After that, catalyst Cu(I)Cl (0.0538 mmol, 0.00538 g) and ligand 2,2′-bipyridine (bpy) (0.108 mmol, 0.017 g) were added to the frozen solution under a N_2_ atmosphere. The reaction started by placing the Schlenk tube into a 65 °C oil bath with constant stirring under protection of a N_2_ atmosphere. The polymerization was monitored by GPC. After 12 h, the reaction was stopped by opening the Schlenk tube to air and diluting the reaction mixture with methanol. Then, the dilute solution was placed into dialysis bags (molecular weight cutoff (MWCO) of 8000–10,000) in order to remove small molecules and impurities. Lastly, with the water changed every 12 h for a week, a white solid of the ABA triblock copolymer was obtained by freeze-drying. A series of ABA triblock copolymers with different polymerization degrees were synthesized by ATRP, and the schematic illustration of the synthesis of ABA triblock copolymer is shown in Scheme 1. Compositions and molecular weight data of the ABA triblock copolymers with different polymerization degrees and different PEG lengths are shown in Table S1.

### 2.5. Transmittance and Surface Tension Analysis of 0.5 mg·mL^−1^ Aqueous Solutions of ABA Copolymer

(1) The ABA copolymers with different PEG lengths and polymerization degrees were dissolved in deionized water to obtain 0.5 mg·mL^−1^ copolymer solutions at ambient temperature. Transmittances of the copolymer aqueous solutions at different temperatures were recorded at 660 nm using UV–visible spectrophotometer (TU-1901, Beijing, China); (2) Aqueous solutions of the copolymers (0.5 mg·mL^−1^) were prepared at ambient temperature, and the pH values of the copolymer solutions were adjusted with 0.5 M HCl or 0.5 M NaOH aqueous solutions. Transmittances of the copolymer aqueous solutions at different pH values were recorded at 660 nm using TU-1901; (3) The surface tension of 0.5 mg·mL^−1^ aqueous solution of the P2 copolymer at different temperatures was recorded by a surface tension analyzer (DCAT 21, GER Dataphysics Instruments Company, Filderstadt, Germany).

### 2.6. Viscosity and Fluorescence Probe Measurements of Thermo-Induced Micellization of ABA Copolymer in Aqueous Solutions

It is well known that the micellization and the critical micelle concentration (CMC) of the stimuli-responsive polymers are usually determined by viscosity measurements and fluorescence probe techniques. (1) The copolymer was dissolved in deionized water to obtain 1 wt % copolymer solution. The pH value of solution was adjusted with 0.5 M HCl or 0.5 M NaOH aqueous solutions. Viscosities of the copolymer with a given pH at different temperatures were measured by SNB-2 digital viscometer (SNB-2, Shanghai Nirun Intelligent Technology Corporation, Shanghai, China); (2) Twenty milligrams of pyrene (Py) was accurately weighed and dissolved with diethyl ether, which was placed into a 10 mL volumetric flask and diluted with diethyl ether to scale, to obtain a stock solution of Py of known concentration (ca. 10^−2^ mol·L^−1^). Py stock solution was diluted to 10^−5^ mol·L^−1^ with diethyl ether before use. The solution (100 µL) was injected into a 10 mL volumetric flask and the ether was evaporated at ambient temperature. Subsequently, a polymer solution of known pH was added to the flask. To ensure solubilization and equilibration, the polymer/probe solution was sonicated for 30 min, and then left at room temperature for more than 12 h. Fluorescence spectra data of the peak intensity ratio (*I*_3_/*I*_1_) (*I*_3_: 384 nm; *I*_1_: 373 nm) for solutions of different pH versus the logarithm of the copolymer concentrations were recorded by time-correlated single-photon-counting fluorescence spectrometer (FLS 920, UK Edinburgh Instruments Company, Edinburgh, UK) at 25 °C. In addition, the excitation wavelength chosen was 333 nm, excitation and emission slit widths were 10 and 2.5 nm, respectively, and scan range was 350–500 nm.

### 2.7. Dynamic Light Scattering (DLS) and Zeta Potential Measurements of ABA in Aqueous Solutions

(1) An aqueous solution of P2 (0.5 mg·mL^−1^) was prepared and the pH value of the solution was adjusted with 0.5 M HCl or 0.5 M NaOH aqueous solutions. The intensity-average hydrodynamic diameter of the copolymer at different temperatures or pH were obtained by Malvern Zetasizer Nano (ZS90, Malvern, England) and all data were averaged over three consecutive runs; (2) An aqueous solution of P2 (1.5 mg·mL^−1^) was prepared and the pH value of solution was adjusted with 0.5 M HCl or 0.5 M NaOH aqueous solutions. Zeta potential of the copolymer solution at different pH values was obtained by a Delsa Nano Zeta Potential analyzer (Delsa Nano C, Beckman Coulter, Inc., Osaka, Japan).

### 2.8. Transmission Electron Microscopy (TEM)

The P2 aqueous solution (1 mg·mL^−1^) was stirred overnight at acid state (pH 5) at 46 °C. The detailed procedure is as follows: a droplet of sample solution was spotted onto the copper grid by microinjector. When the sample was dry, it was stained with phosphotungstic acid (PTA) (1 mg·mL^−1^) whose pH value had been adjusted to 7, and the sample was then subjected to TEM (JEM-2100 instrument from JEOL, Tokyo, Japan). A similar procedure was used to test another sample at basic state (pH 10).

### 2.9. Sol–Gel Transitions of ABA Aqueous Solutions

For the P2 aqueous solutions, the different concentrations (10, 15, 20, 22.5, 25, 27.5, 30, and 32.5 mg·mL^−1^) at pH 7 were heated at a rate of 1 °C·min^−1^ from 25 °C. The sol-to-gel transition temperatures (*T*_sol–gel_) and gel-to-sol temperatures (*T*_gel–sol_) of different concentrations of the P2 solutions are expressed in the phase diagram. In addition, some photos of sols, gels, and sol–gel transition were also obtained. A similar procedure was used to test 30 mg·mL^−1^ P2 aqueous solutions at different pH values.

### 2.10. Application of Gel

P2 (600 mg) and BSA (10 mg) were dissolved in 2 mL distilled water, and the mixture formed a drug-loaded gel at 37 °C. Then, the gel was placed into 100 mL buffer solution of phosphate (pH 7.4) at 32 °C, and the BSA release was measured every 10 min. A similar procedure was used to measure the BSA release content of the drug-loaded gel at different temperatures and pH values.

## 3. Results and Discussion

### 3.1. Synthesis and Characterization of Br–PEG–Br, ABA Copolymer

The macroinitiator Br–PEG–Br was prepared according to Scheme 1, and characterized by ^1^H NMR. Figure S1a is the ^1^H NMR spectra of PEG (A) and Br–PEG–Br (B). The macroinitiator Br–PEG–Br was confirmed by the appearance of the characteristic peak at 1.8 ppm and disappearance of the characteristic peak at 2.4 ppm in the ^1^H NMR spectrum. The peak at 2.4 ppm is attributed to PEG, however, it is replaced by the peak at 1.8 ppm which is attributed to Br–PEG–Br.

The doubly responsive ABA triblock copolymers were synthesized according to Scheme 1. The ABA copolymer was synthesized from the PEG macroinitiator by ATRP of MEO_2_MA, DEAEMA, and OEGMA with a feed molar ratio of 38:3:2 at 65 °C using CuCl/2,2′-bipyridine (bpy) as a catalyst system. With this set-up, copolymers with different PEG lengths and polymerization degrees were synthesized. Figure S1b shows the ^1^H NMR spectra of ABA, the characteristic peak at 3.7 ppm (k) is attributed to PEG of B block, while the characteristic peaks at 4.2 ppm (h), 3.9 ppm (d), 3.6 ppm (i), 3.4 ppm (j), 2.3 ppm (a), 1.9 ppm (e), 1.8 ppm (f), 1.3 ppm (c), 1.1 ppm (g), and 0.9 ppm (b) belong to P(DEAEMA-*co*-MEO_2_MA-*co*-OEGMA) of A block.

The summary of the various triblock compositions and molecular weight data with different PEG lengths and polymerization degrees are shown Table S1. The content of A% (wt %) in the ABA polymers was calculated according to the number-averaged molecular weights of *M*_n_(Br–PEG–Br) and *M*_n_(ABA) as shown in Table S1 [52,53].

### 3.2. Temperature- and pH-Sensitivities of ABA Triblock Copolymers in Aqueous Solution

**Figure 1:**
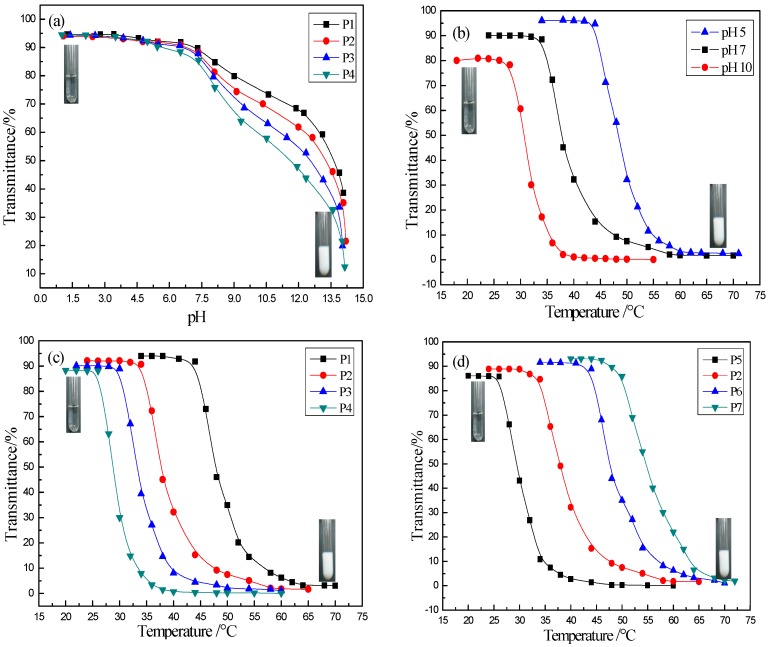
Transmittance curves of: (**a**) P1, P2, P3, P4 in aqueous solutions versus pH at 25 °C; (**b**) P2 in aqueous solution versus temperature at different pH; (**c**) P1, P2, P3, P4 in aqueous solutions versus temperature; and (**d**) P5, P2, P6, P7 in aqueous solutions versus temperature. (0.5 mg·mL^−1^).

Figure 1 shows the transmittance curves of 0.5 mg·mL^−1^ ABA triblock copolymers in aqueous solution versus pH (Figure 1a,b) or temperature (Figure 1c,d), respectively. Firstly, the pH sensitivity of copolymer in aqueous solution can be confirmed by the transmittance curves of the triblock copolymers in aqueous solution versus pH values. Figure 1a shows the transmittance curves of copolymer solutions (0.5 mg·mL^−1^) with different polymerization degrees (P1, P2, P3, and P4) versus pH at 25 °C. It was concluded that the larger the polymerization degree of copolymer, the lower the critical phase transition pH of copolymer. However, because of the small amount of DEAEMA in the P(DEAEMA-*co*-MEO_2_MA-*co*-OEGMA) segment, the pH sensitivity of copolymer is relatively low, so it is difficult to induce the micellization of copolymer in aqueous solution only by adjusting the pH value of solution.

Nevertheless, the effect of the copolymer’s pH sensitivity on its temperature sensitivity is undoubtedly confirmed according to the Figure 1b, where it shows transmittance curves of P2 in aqueous solution versus temperature at pH 5, 7, and 10 values, respectively. It can be seen that the LCST of P2 aqueous solution decreases with increasing pH of the solution. It is well known that the tertiary amine of the DEAEMA segment is protonated at low pH, giving the tertiary amine a positive charge. This results in electrostatic repulsion between the charges, which puts the copolymer chain into a stretched conformation and renders it strongly hydrophilic; this results in the transmittance of the solution being higher. When the solution pH > 8, the deprotonation of the tertiary amine happens, and this causes the copolymer chain to be compressed, thus the copolymer becomes hydrophobic, and transmittance of the solution becomes lower. As a result of that, the LCST of copolymer solution gradually decreases while the pH increases.

At the same time, the temperature sensitivity of the triblock copolymer has been confirmed perfectly in Figure 1c. Transmittance curves of copolymer aqueous solutions versus temperature are shown in Figure 1c. The temperature corresponding to the highest point of the derivation curve of transmittance vs. temperature was defined as the LCST value of the polymer [54]. It can be seen that the change of transmittance versus temperature is regular and that the LCST values of P1, P2, P3, and P4 in aqueous solutions with different polymerization degrees are 47.5, 37.5, 35, and 30 °C, respectively. The higher the polymerization degree of A block, the lower the LCST of ABA triblock copolymer in aqueous solution. When the polymerization degree increases, the hydrophobic interactions among these hydrophobic groups in the P(DEAEMA-*co*-MEO_2_MA-*co*-OEGMA) chains become strong. Thus, the system’s hydrophilic–hydrophobic balance moves towards hydrophobicity, leading to a lower cloud point temperature. As a result, the LCST of the copolymer solution is reduced. Compared with other copolymers, the LCST of the P2 solution is around 37.5 °C, which is very close to human body temperature.

Figure 1d—the transmittance curves of copolymers of different PEG length in aqueous solution versus temperature—shows that the longer the PEG length, the higher the LCST of copolymer aqueous solution. This is because PEG is a hydrophilic segment, and as the PEG length increases, more and more hydrogen bonds between the copolymer chains and the water molecules will be formed. So, a higher temperature is needed to break these hydrogen bonds. Thus, the higher LCST for the copolymer with the longer PEG has appeared, and the LCST values of P5, P6, and P7 in aqueous solutions with different PEG length are 29, 50, and 56 °C, respectively.

**Figure 2:**
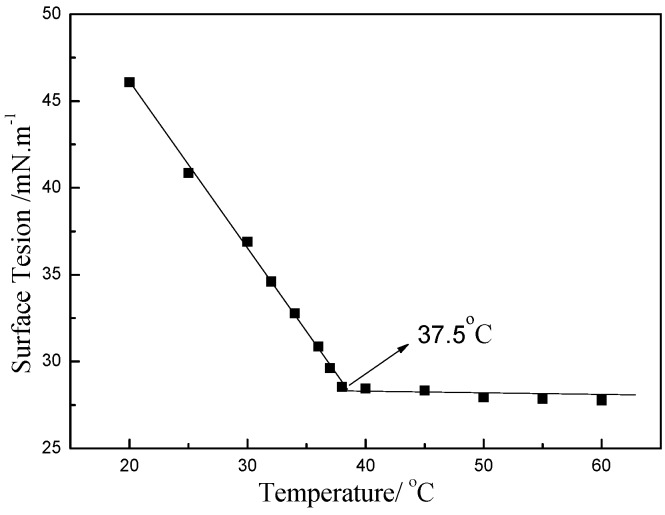
Surface tension curve of P2 aqueous solution (0.5 mg·mL^−1^, pH 7) versus temperature.

The temperature sensitivity of the ABA triblock copolymer can also be compared with surface tension measurements of copolymer aqueous solutions. Because the LCST of P2 aqueous solution (pH 7) was 37.5 °C, which is very close to the body temperature, P2 was chosen as a representative to discuss some properties of the ABA triblock copolymers. Figure 2 shows the curve of the surface tension of the P2 solution versus temperature. We can see that as the temperature increases, the surface tension of the solution becomes lower. This is because the P(DEAEMA-*co*-MEO_2_MA-*co*-OEGMA) blocks in the ABA triblock copolymers increases in hydrophobicity with the rising temperature. The surface tension curve shows an inflection point, which means the copolymer undergoes a phase transition at around 37.5 °C for the P2 aqueous solution. The surface tension decreases slightly before finally approaching a stable value [55].

### 3.3. Viscosity of ABA in Aqueous Solution

**Figure 3:**
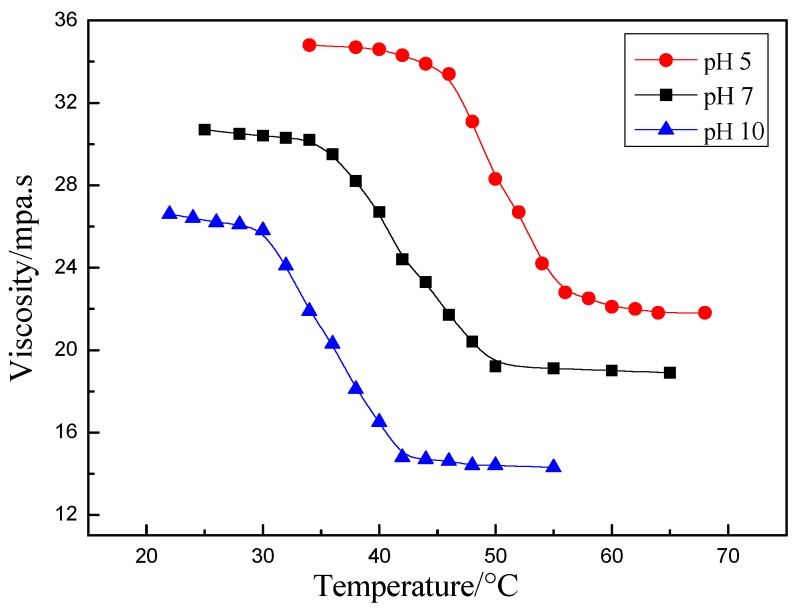
Viscosity curves of 1 wt % P2 in aqueous solution versus temperature at different pH values.

Viscosity curves of the P2 aqueous solution versus temperature at different pH are shown in Figure 3. It can be seen that solution viscosity decreases gradually with the rise in temperature, but solution viscosity declines sharply when temperature is above their LCST at pH 5, 7, and 10, respectively, which is basically consistent with the results of the transmittance measurements. At pH 5, the copolymer chain is loose and hydrophilic, relative motion of the copolymer chains is easy, and the solution viscosity is higher. Change tendency of the solution viscosity at pH 7 and pH 10 is similar with pH 5, but the copolymer chain begins to shrink, becomes dehydrated and hydrophobic, and the solution viscosity becomes lower.

### 3.4. CMC Determination of ABA in Aqueous Solutions by Fluorescence Spectra

The copolymer micelles were also verified using a fluorescence probe technique, with pyrene (Py) employed as the probe. Py is one of the most commonly used fluorescent probes. At room temperature, fluorescence emission spectra of the Py probe appears as five peaks at 373 nm, 378 nm, 384 nm, 392 nm, and 415 nm [56,57]. Moreover, the third (*I*_3_ at 384 nm) and first (*I*_1_ at 373 nm) peaks in the emission spectra are of special interest, as the ratio of the intensities of the third and first peaks can be used to monitor the formation of hydrophobic microdomains [58]. The *I*_3_/*I*_1_ ratio is lower for Py in polar media, but it will increase when polarity of the probe microenvironment decreases.

**Figure 4:**
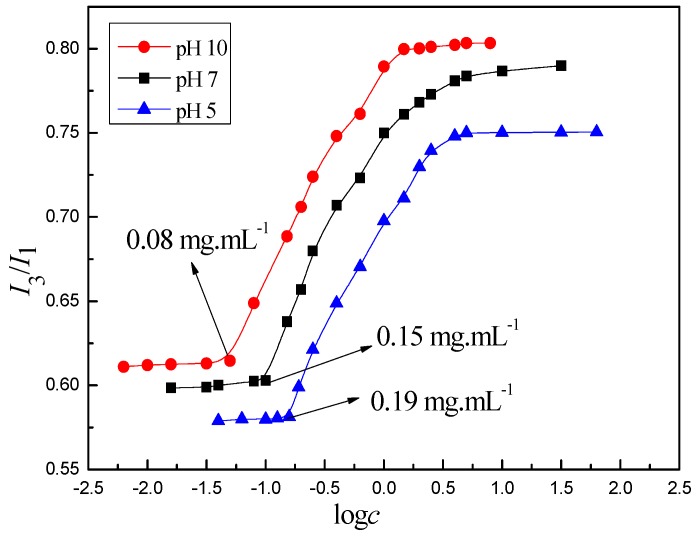
Curves of the *I*_3_/*I*_1_ ratio of pyrene (Py) at 25 °C for various concentrations of P2 aqueous solution at pH 5, 7, and 10.

The curves of the *I*_3_/*I*_1_ ratio of Py at 25 °C for various concentrations (logarithmic scale) of the P2 aqueous solution at different pH values are shown in Figure 4. At lower concentration, the distances between molecules are larger, the polarity of the aqueous solution is higher, so the *I*_3_/*I*_1_ ratios of Py are lower. However, as the concentration of copolymer achieves a suitable value (0.19 mg·mL^−1^, at pH 5; 0.15 mg·mL^−1^, at pH 7; 0.08 mg·mL^−1^, at pH 10), a few micelles are formed by copolymer. When the concentration is higher, the copolymer begins to greatly aggregate, and a great number of micelles are present in the system, which indicates that the Py probe is in a more hydrophobic microenvironment. Thus, the *I*_3_/*I*_1_ ratio increases sharply and remains stable at last. As in the acid state, the protonation of tertiary amine (which belongs to the DEAEMA segment) makes the copolymer chain stretched, so the copolymer is hydrophilic. Thus, it is difficult to form the hydrophobic microenvironment, and requires copolymer higher concentrations to form the micelles. Oppositely, copolymer becomes more hydrophobic due to the deprotonation of tertiary amine in its basic state, which leads to formation of a large hydrophobic microenvironment. Therefore, only at a low concentration are many micelles easily formed by copolymer. In summary, the critical micelle concentration (CMC) in acid state is higher than that in neutral solution and basic state. It can be seen that the pH sensitivity has a great influence on the CMC of copolymer in aqueous solution.

### 3.5. Aggregate Behaviors of ABA in Aqueous Solutions

**Figure 5:**
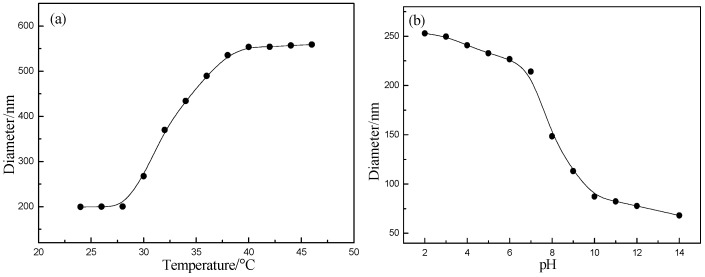
The aggregate particle diameters in P2 aqueous solution as a function of temperature (**a**) and solution pH (**b**) at 25 °C (0.5 mg·mL^−1^).

To study aggregate behavior changes of the polymer with temperature, the aggregate particle diameters in P2 aqueous solution (0.5 mg·mL^−1^) studied by DLS at different temperatures are shown in Figure 5a. At low temperature, the diameter is around 202 nm, but at higher temperature (around 37.5 °C) a dramatic increase in aggregate particle diameter occurs. However, the diameter trends toward a stable value when the temperature is above the LCST, which is consistent with the transmittance measurements. This phenomenon can be attributed to the temperature-induced self-assembly of the ABA triblock copolymers [59].

Figure 5b shows the curve of the aggregate diameters in P2 aqueous solution (0.5 mg·mL^−1^) versus pH values. At low pH values, the tertiary amine on the PDEAEMA segment is protonated and the protonated tertiary amine has a positive charge, causing electrostatic repulsion between the charges and a stretched formation of the copolymer chain. This makes the copolymer swollen and hydrophilic, resulting in a larger diameter. Due to the deprotonation of the tertiary amine in basic medium, the copolymer becomes dehydrated, compact, and more hydrophobic. As a result of that, a dramatic decrease in aggregate particle diameter occurs as the pH changes from 7.4 to 10.5. However, when the pH was above 11, the diameter achieved a stable value.

### 3.6. Charge Properties of ABA in Aqueous Solution

**Figure 6:**
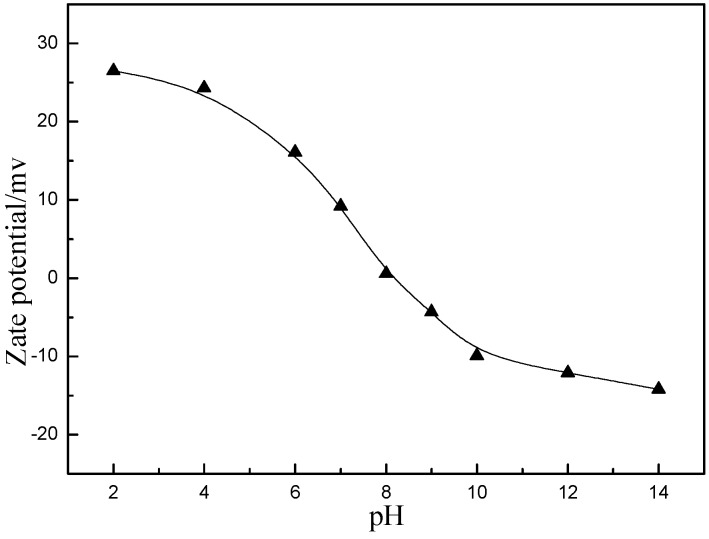
Zeta potentials of P2 aqueous solution (1.5 mg·mL^−1^) versus pH at 25 °C.

The charge property along the copolymer chains can be usually determined by measurement of zeta potentials. Figure 6 shows the zeta potentials of P2 aqueous solution (0.5 mg·mL^−1^) versus pH values at 25 °C. In this case, the copolymer is positively charged in acid medium, and negatively charged when it is in basic medium. In addition, the copolymer is nearly uncharged at pH 7.4. As mentioned above, the zeta potential is the ultimate and reasonable explanation for the characters of the copolymer aqueous solutions at different pH values, such as transmittance, viscosity, aggregate particle diameters, and the CMC. In other words, changes of the charge properties along with the copolymer chains play a dominating role for pH-responsive behaviors [60,61].

### 3.7. TEM Images of the Temperature-Induced Micelles

**Figure 7:**
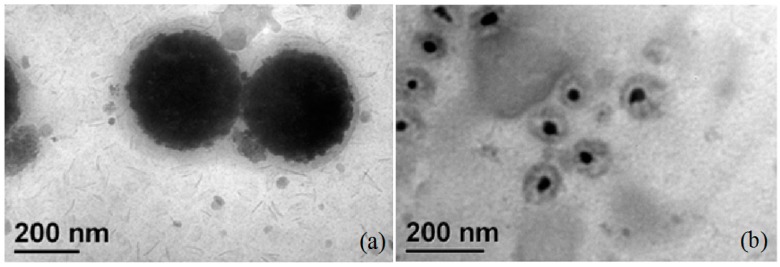
TEM images of micelles of P2 at 46 °C in acid medium (**a**) and basic medium (**b**).

**Scheme 2:**
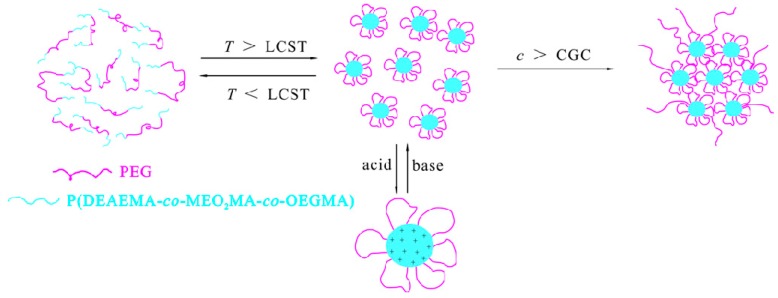
Micellization and gelation process of the ABA triblock copolymer.

Figure 7 shows the TEM images of micelles of P2 at 46 °C. It is well known that when the temperature of a copolymer solution is higher than its LCST, the triblock copolymer chains form large and stable core–shell micelles with compact P(DEAEMA-*co*-MEO_2_MA-*co*-OEGMA) cores and loose PEG shells. As shown in Figure 7a, in acid medium, the protonation of the tertiary amine, which belongs to the P(DEAEMA-*co*-MEO_2_MA-*co*-OEGMA) block, makes the core more swollen. As a result of that, the protonated size of the core–shell micelles are bigger than before protonation—the diameter is about 500 nm. Nevertheless, as shown in Figure 7b, the deprotonation of tertiary amine in basic medium leads to the cores being more collapsed, so the core–shell micelles become much smaller, and the diameter is just about 100 nm. What the TEM images in Figure 7 have shown are consistent with the influence of pH-sensitivity on aggregate particle diameters in Figure 5b. In addition, the reason for a little difference in diameters between TEM images and DLS data is due to the test conditions: the samples used for TEM were dry on a copper grid, while the samples of DLS were dissolved in aqueous solution. For the latter, there are presenting hydrogen bonds between copolymer and water molecules, which makes the diameters a little bigger than that on copper grid for testing by TEM. Furthermore, the formation process of a temperature-induced core–shell micelle and the great influence of pH on temperature-induced micelles in aqueous solution are drawn in Scheme 2.

### 3.8. Temperature, Concentration, and pH Effects on Sol–Gel Transition of ABA in Aqueous Solution

**Figure 8:**
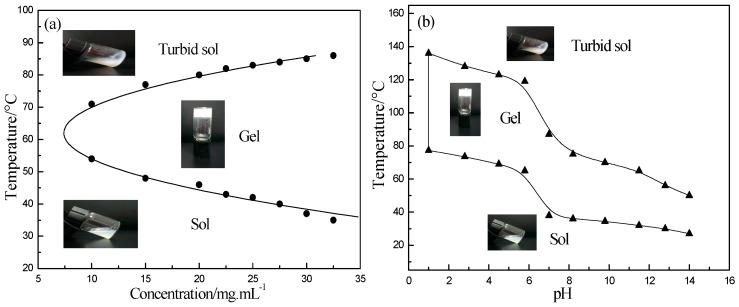
The temperature induced sol–gel phase diagrams of P2 aqueous solution (**a**) and 30 mg·mL^−1^ P2 aqueous solution at different pH (**b**).

The temperature-induced sol–gel transition phase diagram for various concentrations of P2 aqueous solution is shown in Figure 8a; the critical gelation concentration (CGC) of the copolymer solution is about 8 mg·mL^−1^. When temperature is above the LCST, a few micelles are formed, and with the temperature rising further, more and more micelles are formed. While the copolymer concentration is above the CGC, a 3-dimensional micellar network is formed, in which the central block forms bridges among neighboring micelles, and the free-flowing solution is transformed into a micellar gel [8]. In addition, because of a high temperature, the gel has been broken and copolymer chains begin to aggregate further to form the turbid sol. Figure 8a indicates that when the copolymer concentrations increase from 10 to 32.5 mg·mL^−1^, the sol-to-gel transition temperatures (*T*_sol–gel_) in aqueous solution decrease from 52 to 33 °C, but their gel-to-sol transition temperatures (*T*_gel–sol_) increase from 71 to 86 °C. In other words, the lower the copolymer concentration is, the higher its *T*_sol–gel_ and the lower the *T*_gel–sol_ are. On the contrary, the higher the copolymer concentration is, the lower its *T*_sol–gel_ and the higher the *T*_gel–sol_ are. The higher the concentration of the copolymer aqueous solution is, the more compact the 3-dimensional micellar network is, which makes the copolymer solution form the gel easily at a lower temperature. However, due to the more compact and stable 3-dimensional micellar network, to change the gel to a turbid sol requires a higher temperature, compared with that at the lower copolymer concentration.

Figure 8b shows the sol–gel phase diagram of P2 aqueous solution (30 mg·mL^−1^) induced by temperature at different pH values. It can be seen that when the pH value changes from 1 to 14, the *T*_sol–gel_ decreases from 77.3 to 27 °C, and *T*_gel–sol_ decreases from 136 to 50 °C, indicating that both *T*_sol–gel_ and *T*_gel–sol_ decrease with increasing values of pH. With the increasing of pH values, instead of the protonation of tertiary amine in acid medium, the tertiary amine is deprotonated in basic medium, thus, the copolymer chain becomes compact and more hydrophobic, so it is easy to form a gel at a lower temperature. Furthermore, in basic medium, due to the great hydrophobicity, less power is needed to break a gel to form a turbid sol, thus the *T*_gel–sol_ is lower than that in acid medium.

**Figure 9:**
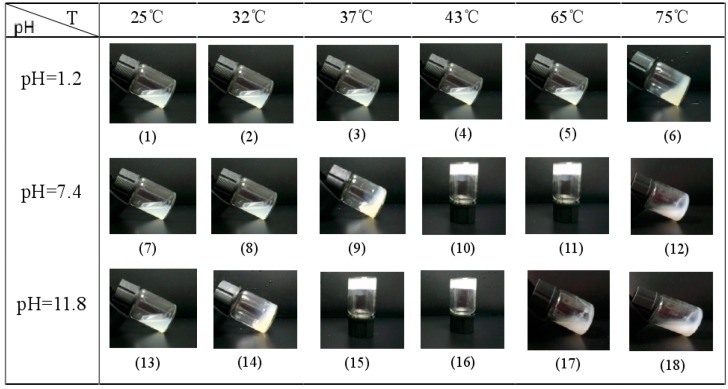
Photo of P2 aqueous solution at 25, 32, 37, 43, 65, and 75 °C (30 mg·mL^−1^, at pH 1.2, 7.4, and 11.8).

In order to introduce the process of the sol–gel transitions visually, some photos of gels in different conditions are shown in Figure 9 [12]. The P2 aqueous solution (30 mg·mL^−1^) is still a free-flowing liquid at 25 °C when the pH is 1.2, 7.4, and 11.8, respectively. It turns into a weak gel at 32 °C only at pH 11.8. This indicates that the formation of gel in basic medium is easier and the *T*_sol–gel_ is lower. Further increasing the temperature to 37 °C, a weak gel is formed at pH 7.4, and a free-standing gel is formed when the pH is 11.8. At 43 °C, the free-standing gel is formed at pH 7.4, and free-standing gel still exists at pH 11.8. Furthermore, the free-standing gel remains unchanged at pH 7.4, but it turns into a turbid sol at pH 11.8 when the temperature reaches to 65 °C. Lastly, at 75 °C, a weak gel is eventually formed in acid medium (at pH 1.2), the free-standing gel turns into a turbid sol at pH 7.4, and the turbid sol reverts to its original state at pH 11.8. In summary, what has been shown in Figure 9 is consistent with the content of two phase diagrams (Figure 8) which have shown the great influence of temperature and pH on the sol–gel transitions. All in all, the temperature- and pH-induced gelation processes described above can be interpreted by Scheme 2.

### 3.9. BSA Release of BSA-Loaded Micellar Gel which Formed by ABA in Buffer Solution

BSA is the most abundant globular protein in the plasma. This protein is commonly used as a model protein due to its medicinal importance, low cost, ready availability, stability, water solubility, and structural similarity with human serum albumin (HSA). Various endogenous and exogenous ligands are transported by BSA. All of these features make BSA one of the best models to understand the physicochemical basis of polymer–protein interactions [62].

The calibration curve between concentration and absorbance (Abs) of BSA is shown in Figure S2. When the BSA concentration in aqueous solution ranges from 0.01 to 1 mg·mL^−1^, there will be a linear relationship between BSA concentration and Abs, which could be performed through *c* (mg·mL^−1^) = 1.14286A − 0.10202. Then, the cumulative release is given by: cumulative release (%) = (*W*_t_/*W*) × 100, where *W*_t_ is the cumulative weight of drug in release medium which has been freed from the system at time t, and *W* is the original weight of BSA.

**Figure 10:**
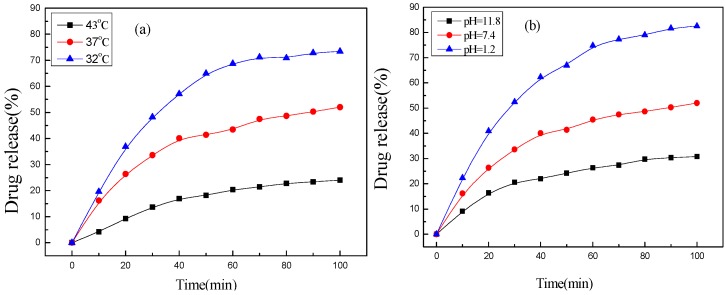
The cumulative release curves of bovine serum albumin (BSA) at different temperatures (**a**) and different pH (**b**).

The release curves of BSA-loaded micellar gel (30 mg·mL^−1^) versus time are shown in Figure 10. In Figure 10a, the BSA release curves at different temperatures, the higher the temperature is, the slower the BSA release is. In ABA gel, the BSA release reaches 75% in 100 min at 32 °C, but the BSA release is only close to 55% at 37 °C, while the BSA release percentage is much smaller at 43 °C. The reasons are as follows. At the lower temperature (at 32 °C), the structure of BSA-loaded micellar gel is broken, copolymer chains begin to stretch and become looser, so BSA is easily released from the micellar gel. At a higher temperature (at 43 °C), the gel is more stable, copolymer chains begin to shrink and become more compact, so it is harder to release the BSA.

Furthermore, the medium’s pH also has a big effect on the BSA release from the micellar gel. Especially, artificial gastric juice (pH 1.2) and intestinal juice (pH 7.4) were used as the buffer solution at 37 °C to make the test more relevant and convincing. Figure 10b shows the release curves in ABA gel at different pH when temperature is 37 °C; the higher the pH is, the slower the BSA release is. The BSA release is so large that it nearly reaches 85% in artificial gastric juice (pH 1.2) in 100 min, but the BSA release is only close to 51% in intestinal juice (pH 7.4). With further increasing pH (to pH 11.8), the BSA release becomes much slower and is only 35%. As we all know, the protonation of tertiary amine in artificial gastric juice (pH 1.2) leads to copolymer chains becoming stretched, thus the BSA-loaded micellar gel is disintegrated greatly, and BSA is released faster than in artificial intestinal juice (pH 7.4). Furthermore, in basic medium, the copolymer chains become more compact in the BSA-loaded gel, which is attributed to the deprotonation of tertiary amine, so it is harder to release the BSA.

## 4. Conclusions

Novel temperature- and pH-sensitive ABA triblock copolymers were synthesized by ATRP. The LCST of the synthesized copolymers decreased with increasing degree of polymerization of the P(DEAEMA-*co-*MEO_2_MA-*co*-OEGMA) block and decreasing the length of the PEG chain. The LCST of P2 aqueous solution was 37.5 °C, which is very close to the body temperature. CMC of the P2 aqueous solution at pH 7 was 0.15 mg·mL^−1^, and its CMC was decreased with an increase in pH. *T*_sol–gel_, *T*_gel–sol_, and gel strength can be tuned by varying the solution pH and concentration of the copolymer aqueous solution, and the *T*_sol–gel_ increased with decreasing copolymer concentration or solution pH. BSA release experiments show that BSA cumulative release decreases with increasing temperature and solution pH, respectively.

## Supplementary Materials

The following are available online at www.mdpi.com/2073-4360/8/11/367/s1.
