# Supplementary material for: Synthesis of Novel Temperature- and pH-Sensitive ABA Triblock Copolymers P(DEAEMA-co-MEO2MA-co-OEGMA)-b-PEG-b-P(DEAEMA-co-MEO2MA-co-OEGMA): Micellization, Sol–Gel Transitions, and Sustained BSA Release
Source: Polymers (Basel). 2016 Nov 11;8(11):367. doi: 10.3390/polym8110367 (PMC6431942; doi:10.3390/polym8110367)
Supplement: Supplementary file 1 [file polymers-08-00367-s001.pdf]

Supplementary Materials: Synthesis of Novel Temperature- and pH-Sensitive ABA Triblock Copolymers P(DEAEMA-*co*-MEO2MA-*co*-OEGMA)-*b*-PEG-*b*-P(DEAEMA-*co*-MEO2MA-*co*-OEGMA): Micellization, Sol–Gel Transitions, and Sustained BSA Release

Yanan Han, Shouxin Liu, Hongguang Mao, Lei Tian and Wenyan Ning

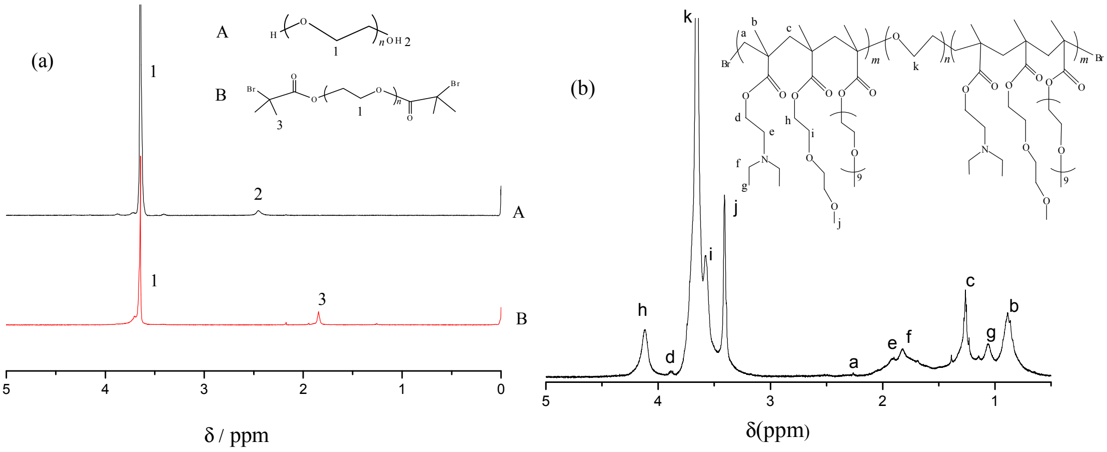

Figure S1. ^{1}H NMR spectra of: (**a**) poly(ethylene glycol) (PEG) (A), Br–PEG–Br (B); and (**b**) ABA.

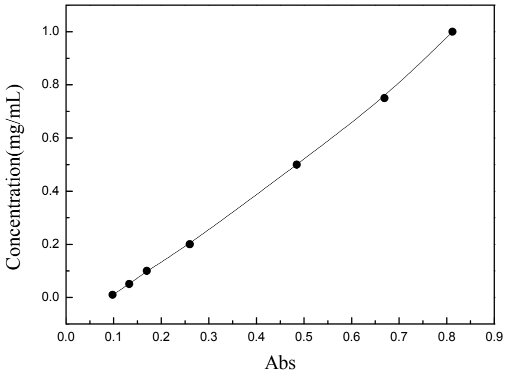

Figure S2. The calibration curve of concentration versus absorbance (Abs) of BSA in buffer solution (pH 7.4).

Table S1. Characterization of copolymers.

ABA	PD of A block	M_n (Br-PEG-Br)	M_n (ABA) [a]	PDI [a]	A% (wt %)	LCST/°C
P1:A50BA50	50	4,659	27,687	1.19	42	47.5
P2:A100BA100	100	4,659	44,783	1.18	45	37.5
P3:A150BA150	150	4,659	63,480	1.11	46	35
P4:A200BA200	200	4,659	88,593	1.01	47	30
P5:A100BA100	100	2,159	43,289	1.21	48	29
P6:A100BA100	100	8,359	48,563	1.11	42	50
P7:A100BA100	100	10,583	50,029	1.01	40	56

[a] Determined by gel permeation chromatography (GPC).
